# Comparative Study of Adverse Drug Reactions Associated with Filgrastim and Pegfilgrastim Using the EudraVigilance Database

**DOI:** 10.3390/biology11020340

**Published:** 2022-02-21

**Authors:** Shruti Rastogi, Vivekanandan Kalaiselvan, Yousef A. Bin Jardan, Saima Zameer, Maryam Sarwat

**Affiliations:** 1 Amity Institute of Pharmacy, Amity University, Noida 201313, India; shruti.ipc@gov.in; 2 Indian Pharmacopoeia Commission, Ministry of Health & Family Welfare, Government of India, Ghaziabad 201002, India; kalaiselvan.ipc@gov.in; 3 Department of Pharmaceutics, College of Pharmacy, King Saud University, Riyadh 11451, Saudi Arabia; ybinjardan@ksu.edu.sa; 4 Department of Neurodegenerative Science, Van Andel Institute, Grand Rapids, MI 49503, USA; saima.zameer@vai.org

**Keywords:** adverse drug reaction, EudraVigilance, neupogen, safety, filgrastim, pegfilgrastim

## Abstract

**Simple Summary:**

The most commonly reported adverse drug reactions (ADRs) related to filgrastim (FIL) and pegfilgrastim (PEG-F) were obtained and analyzed from the European EudraVigilance (EV) database. Frequently reported ADRs for FIL and PEG-F are pyrexia, bone pain, back pain, neutropenia and febrile neutropenia. No statistical difference in the probability of bone pain between FIL and PEG-F was observed. To further depict the safety of FIL and PEG-F, there is a further need to examine the real-life data.

**Abstract:**

The primary prophylaxis with filgrastim (FIL) and pegfilgrastim (PEG-F) is recommended to decrease the severity of chemotherapy-induced neutropenia (CIN). The commonly reported adverse drug reactions (ADRs) with FIL and PEG-F is bone pain. ADRs pertaining to FIL and PEG-F were extracted from the European EudraVigilance (EV) database. The Individual Case Safety Reports (ICSRs) obtained from EV database that reported FIL and PEG-F as the suspected drug were analyzed. Registered ADRs (from the groups “General disorders and administration site conditions”, “Blood and lymphatic system disorders”, “Musculoskeletal and connective tissue disorders” and “Investigations”) for FIL and PEG-F were collected from EV database from 2007 to 5 June 2021. The reporting odds ratio (ROR) was used to calculate ICSRs with most common ADRs related to FIL and PEG-F. A total of 17,403 ICSRs described the incidence of most common ADRs of FIL and PEG-F. The commonly reported ADRs for both drugs were pyrexia, bone pain, back pain, neutropenia and febrile neutropenia. The odds ratio of ICSRs belonging to the System Organ Class (SOC) “Investigations” (ROR 1.01 (CI 0.93–1.10)) revealed no significant difference in FIL and PEG-F. However, for the SOCs (General disorders and administration site conditions” and “Musculoskeletal and connective tissue disorders” ((ROR 1.14 (CI 1.06–1.21); ROR 1.21 (CI 1.18–1.32), respectively), an increased reporting probability with PEG-F was found. The authors reported a lower reporting probability for the SOC “Blood and lymphatic system disorders” for FIL versus PEG-F (ROR 0.75 (CI 0.70–0.80)). Our results have demonstrated that the occurrence of bone pain was similar with FIL and PEG-F. For the incidence of pyrexia and back pain, PEG-F was associated with a higher reporting probability as compared to FIL. However, the incidence of neutropenia and febrile neutropenia was higher in FIL compared to PEG-F. Further evaluation of data from real life is needed.

## 1. Introduction

Neutropenia and its complications, including febrile neutropenia (FN), mainly caused by cancer chemotherapy, can result in sepsis and infection, leading to oncologic emergency [1,2]. FN leads to a significant neutrophilic reduction accompanied by fever and is defined internationally as “an oral temperature of >38.3 °C or two consecutive readings of >38° for 2 h associated with an absolute neutrophil count (ANC) of less than 0.5 × 10^9^/L or expected to fall below <0.5 × 10^9^/L” [3]. The prevention and correct management of FN is important because the rate of major complications (e.g., acute renal failure, hypotension, and heart failure) is approximately 25% to 30% and FN mortality rate ranges from 9% to 12% [4]. The severe sepsis and hospital mortality may also be as high as 50%. In chemotherapy patients, FN results in decreasing dose, treatment delay or treatment cessation [5]. In America, the mean length of hospitalization stay for solid tumor patients was 8–11 days and for lymphoma patients was 10–11 days, thereby imposing an economic burden on patients [6,7].

Granulocyte colony-stimulating factor (G-CSF) is a glycoprotein responsible for the proliferation, differentiation and activation of hematopoietic blood cells [1,8]. Primary prophylaxis with G-CSF is recommended by international guidelines to reduce the frequency, severity, duration and risk of chemotherapy-induced neutropenia when the risk associated with FN is ≥20% [1,3]. In patients with 10% to 20% risk of FN, G-CSF must be indicated in patients with age more than 65 years, hepatic or renal dysfunction, persistent neutropenia, coexisting morbidities or prior episodes of FN. For patients with FN < 10%, no G-CSF prophylaxis is recommended [3,9,10]. The G-CSFs widely used are short-acting G-CSF (filgrastim (FIL): NEUPOGEN^®^, lenograstim, tbo-filgrastim and filgrastim biosimilars (FIL biosimilars)) and long-acting G-CSFs (pegfilgrastim (PEG-FIL): Neulasta^®^ and lipegfilgrastim).

The first G-CSF to be granted approval in the U.S.A. and Europe was FIL in 1991 [11]. FIL stimulates the bone marrow to produce white blood cells (WBCs), enhances the functions of WBCs and decreases the duration and severity of neutropenia [12]. PEG-F is produced by covalently adding a 20 kDa polyethylene glycol moiety to the N-terminal methionyl residue of FIL. Thus, PEG-F tends to have a higher serum half-life and subsequently attains a higher plasma concentration [13]. Numerous trials have shown that a single dose of 6 mg PEG-F was equally effective as a daily dose of FIL (5 mcg/kg) to reduce the length of neutropenia [14,15,16,17,18]. Lipegfilgrastim, approved by the European Medicine Agency (EMA) in 2013, is a conjugate of FIL with a single polyethylene glycol molecule and approved to reduce the duration and incidence of neutropenia in chemotherapy patients [19].

Numerous studies have shown the comparable safety and efficacy of FIL and PEG-F. The frequently reported ADRs attributed to of FIL are bone pain, rash, allergic reactions, headache and splenomegaly. Among the reported ADRs, bone pain is consistently reported across all patient population [20,21]. However, ADRs associated with PEG-F includes bone pain and pain in the extremities [22]. The authors recently reported the pharmacovigilance data for cases of filgrastim and biosimilar filgrastim using VigiBase^®^, a WHO pharmacovigilance database [23] that confirmed that bone pain is most commonly associated an ADR of FIL innovator. However, some questions remain to be answered especially as it concernsthe relatively low reporting in Europe in the VigiBase^®^ database. Therefore, this paper aims to identify and describe ADRs related with FIL and PEG-F using the European ADR database, EudraVigilance (EV) of the European Medicine Agency (EMA). A statistical analysis is performed and odds ratio with 95% confidence interval (CI) is calculated using the reporting odds ratio (ROR) method. This study is extremely relevant as the increase in patients exposed to chemotherapy may lead to an increase in the potentially life-threatening condition of neutropenia.

## 2. Methodology

### 2.1. Data Source

Data on ICSRs with FIL and PEG-F as the suspected drug were obtained from the European pharmacovigilance database (EudraVigilance; EV database) (www.adrreports.eu, accessed on 30 November 2021 ) from 2007 to 5 June 2021. The EV database is an interface designed to facilitate the electronic reporting of suspected ADRs and analysis of their data. The ADR reports are electronically submitted to the EV database by national medicine regulatory authorities and by Marketing Authorization Holders (MAH) [24]. The database was first launched and became operational in December 2001.

The drugs in the database are coded as per the EV database medicinal product dictionary. ADRs are coded using terms in Medical Dictionary for Regulatory Activities (MedDRA), a standardized medical terminology that helps to share the regulatory information for medical products internationally. There are five hierarchies to MedDRA for the analysis of ADR reports. The highest level of the MedDRA terminology has the lowest specificity and enables the analysis of aggregate data. In contrast, the lowest level of MedDRA terminology has the highest specificity and enables the analysis at an individual level. The Lowest Level Term (LLT) was derived from the most frequently reported ADRs by patients or healthcare professionals (HCPs). Each LLT is linked to its specific preferred term (PT) that is a descriptor term. There is no limit to the number of LLTs that are linked to PT. However, a PT must be linked to at least one LLT. These PT have superordinate descriptors as High-Level Term (HLTs), which are subordinate to “High Level Group Terms” (HLGTs). The HLGTs are linked to one System Organ Classes (SOCs). The SOC is the highest level of hierarchy and comprises the etiology, manifestation site and purpose and thus are the aggregate level of analysis [25].

### 2.2. Data Selection

This is a retrospective, observational, and pharmacovigilant study performed using the EV database. In the database, the valid ICSRs, which reported either FIL or PEG-F as a “suspected/interacting” drug, were identified. For each drug, the cases were retrieved using standardized MedDRA queries (SMQ). The ADRs were described in terms of the SOC term and PT term. The SOCs described were “General disorders and administration site conditions”, “Blood and lymphatic system disorders”, “Musculoskeletal and connective tissue disorders” and “Investigations”. The top reported SOCs and their subsequent top reported PTs were recognized by summarizing the suitable PT or by the application of the appropriate SMQs.

### 2.3. Data Analyses

Using the EV database, the following data were extracted from the ADR reports: (1) patient demographics (patient age and sex); (2) ADR information (outcome and seriousness); (3) administrative information (reporter qualification and source country for regulatory purposes); and (4) medication information (co-reported active ingredients, suspected drugs other than FIL or PEG-F).

The ADR was considered as serious if it resulted in “death”, is “life-threatening”, requires “hospitalization” or “prolonged existing hospitalization”, result in significant “disabilities” and/or is a “congenital anomaly/birth defect” [26]. The outcome of the ADR was classified based on the recovery pattern. These ADR were reported for ADRs belonging to the SOCs “General disorders and administration site conditions”, “Musculoskeletal and connective tissue disorders”, “Blood and lymphatic system disorders” and “Investigations”. The analyses in EV database were electronically based without any manual assessment of the cases.

The primary outcome of interest was the top reported ADRs: pyrexia, neutropenia, febrile neutropenia, bone pain, back pain, and arthralgia. These ADRs were identified by the MedDRA^®^ PT term “Pyrexia”, “Neutropenia”, “Febrile Neutropenia”, “Back Pain”and “Bone Pain”. In the secondary analysis, other potential safety signals, including “drug ineffectiveness”, “arthralgia” and “thrombocytopenia”, were investigated.

The odds ratio and its 95% CI were calculated using ROR method and chi-square test was calculated to compare ADRs “Pyrexia”, “Neutropenia”, “Febrile Neutropenia”, “Back Pain” and “Bone Pain”, between FIL and PEG-F, using FIL as the reference drug. Data analysis was conducted using GraphPad prism 6.0 software (GraphPad Software, San Diego, CA, USA).

## 3. Results

**Figure 1:**
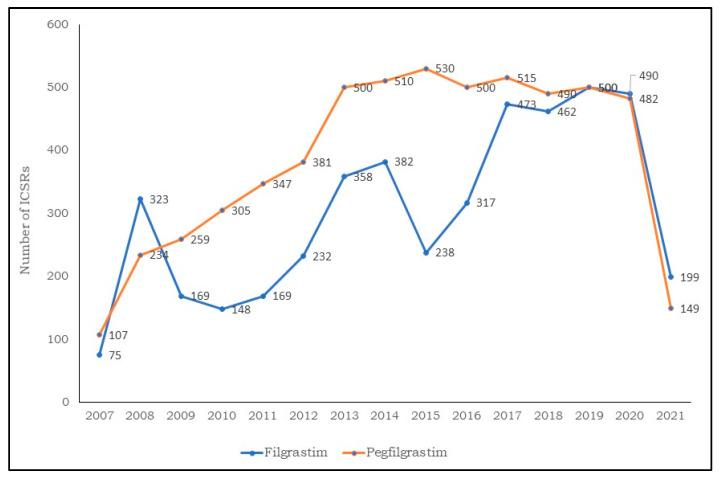
Distribution of individual case safety reports having filgrastim and pegfilgrastim as suspect drugs by year (2007–2021).

We identified 17,403 ICRS with FIL or PEG-F as the drug suspects. Of these, 7091 ICSRs were reported for FIL and 10,312 ICSRs for PEG-F were sent to the EV database. As depicted in Figure 1, a sharp increase in the number of cases was observed in 2013 for FIL and in 2015 for PEG-F, although the data relating to 2021 is incomplete (updated 5 June 2021).

**Figure 2:**
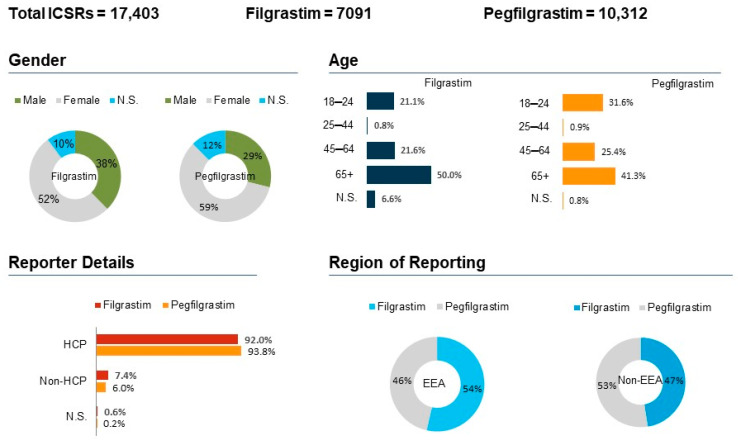
Spontaneous reports having the Filgrastim or the Pegfilgrastim as suspect drugs sent through the EudraVigilance database. ICSRs = Individual Case Safety Reports; EEA = European Economic Area; Non-EEA = Non European Economic Area; HCP = Healthcare Professionals; Non-HCP = Non Healthcare Professionals; N.S. = Not specified.

The characteristics of the ICSRs on the basis of the outcome of interest is described in Figure 2. The age group of 18–64 years experienced most ADRs in FIL (*n* = 3542; 50%) and PEG-F (*n* = 4254; 41.3%), among which the majority were females (52.1% FIL and 58.6% PEG-F). For FIL, the majority of the reports were from Italy (23.9%), France (16.0%), Germany (15.2%) and the United Kingdom (11.5%). Conversely, for PEG-F, the majority of the reports were from Germany (27.6%), followed by Netherlands (27.5%); reports from France and Italy were less than 10%. Regarding distribution by seriousness, 84.3% FIL ICSRs and 93.6% PEG-F ICSRs were considered as serious. Moreover, 14.7% FIL ICSRs and 6.3% PEG-F ICSRs were considered as non-serious. HCPs were the primary reporter of ADRs (FIL: *n* = 6525; 92%; PEG-F: *n* = 9672; 93.8%), while the non-European Economic Area (EEA) countries were the primary source (FIL: *n* = 3901; 55%; PEG-F: *n* = 6294; 61%).

Table 1 describes the frequently reported PTs for FIL and PEG-F. The top three reported ADRs for FIL include neutropenia (8.1%), pyrexia (7.8%) and febrile neutropenia (7.4%). Additionally, the top reported ADRs for PEG-F include febrile neutropenia (13.4%), neutropenia (10.9%) and bone pain (6.8%).

**Figure 3:**
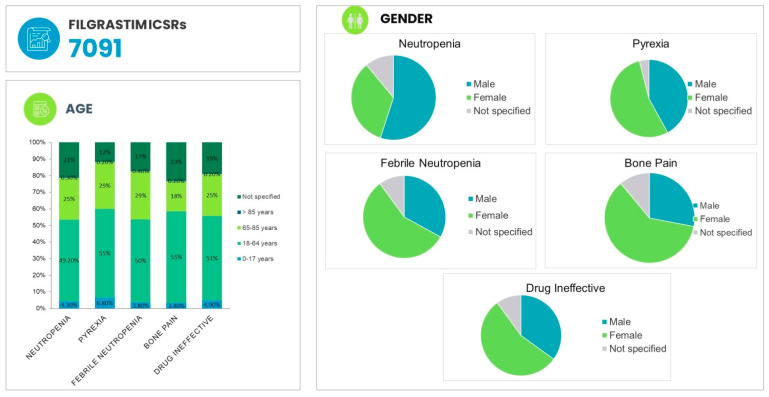
Patient demographic information of adverse events associated with filgrastim.

**Figure 4:**
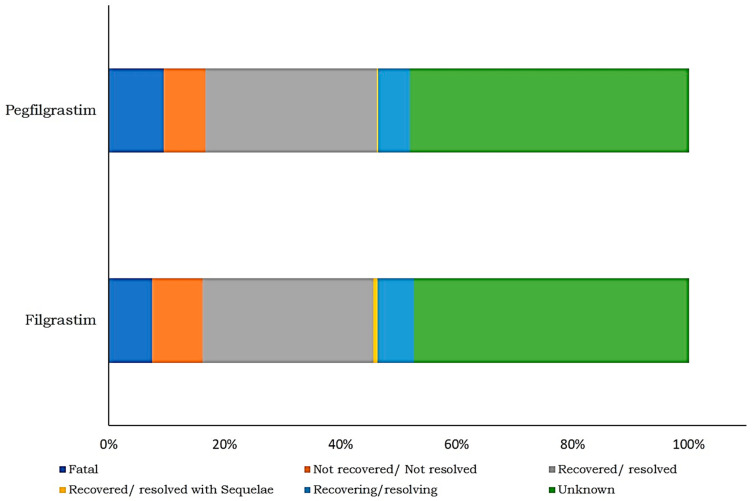
Distribution of filgrastim- and pegfilgrastim-induced ADRs by outcome.

The patient demographic information of ADRs associated with FIL are provided in Figure 3.The most common ADR associated in with FIL in the age group of 18–64 years is pyrexia (55.5%), followed by bone pain (54.8%) and thrombocytopenia (52.5%). Regarding the outcome of ADRs, in the majority of cases, the result was unknown for FIL (47.5%) and for PEG-F (48.2%). However, 29% of the outcome was recovered/resolved in both FIL and PEG-F. In addition, 7.5% of FIL ADRs and 9.5% PEG-F ADRs were fatal (Figure 4).

The disproportionality analysis is provided in Table 2. PEG-F is associated with a high reporting of spontaneous data with ADRs belonging to the SOCs “General disorders and administration site conditions” (ROR 1.14, 95% CI 1.06–1.21) and “Musculoskeletal and connective tissue disorders” (ROR 1.21, 95% CI 1.18–1.32). Instead, the reporting probability of ICSRs was low for ICSRs related to ADRs with the SOC “Blood and lymphatic system disorders” for FIL versus PEG-F (ROR 0.75, 95% CI 0.70–0.80). No substantial difference among FIL and PEG-F was observed for the ROR of ICSRs with ADRs belonging to the SOC “Investigations” (ROR 1.01, 95% CI 0.93–1.10). For specific ADRs, a higher reporting was found for the PT terms “Pyrexia” and “Back Pain” for FIL versus PEG-F (ROR 1.16, 95% CI 1.04–1.30; ROR 1.87, 95% CI 1.57–2.22, respectively). However, the ADRs reporting low probability were neutropenia and febrile neutropenia for FIL versus PEG-F (ROR 0.72, 95% CI 0.65–0.80; ROR 0.51, 95% CI 0.46–0.57, respectively). No difference in the odds ratio among FIL and PEG-F was observed with the ADR “Bone Pain” (ROR 0.72, 95% CI 0.65–0.80). Table 3 provides the ROR belonging to the top reported ADRs for the comparison of FIL versus PEG-F.

## 4. Discussion

FN is the most serious complication associated with cancer chemotherapy, leading to infection and sepsis [1]. It affects quality of life by predisposing patients to long-term treatment and prolonged hospitalization [27]. G-CSF, including FIL and PEG-F, are a choice of drugs for the prophylaxis of chemotherapy-induced FN and numerous clinical trials, and daily practice has demonstrated their efficacy [1,3,28]. Indeed, previous published data comparing FIL and PEG-F have concluded similar efficacy between the two G-CSFs and the need for fewer PEG-F injections can improve the quality of life of the patient and their family.

The present article discusses the post-marketing surveillance of biotherapeutics, i.e., FIL and PEG-F. Unlike chemical entities, biotherapeutics are produced in living organisms, larger in size, and have structural and manufacturing complexities. Biotherapeutics also require adequate temperature for storage to prevent any degradation of the products. The market authorization of biotherapeutics is granted based on abbreviated pathway and ADRs previously not known during clinical trials may appear in a post-marketing setting. To our knowledge, this is the first study performed for the analysis of spontaneous ADRs of FIL and PEG-F through the EV database. The choice to evaluate the top reported ADRs was driven by the scientific evidence to understand the association between FIL and PEG-F [29]. Additionally, with the patent expiry of the FIL innovator in Europe in 2006, several biosimilars have been approved. Interestingly, the data submitted to VigiBase^®^ were limited. Therefore, the authors chose to work on reports submitted to the European adverse drug reaction EV database to study the ADR reports of FIL and PEG-F.

In our study, we found 17,403 ICSRs reporting FIL or PEG-F as a suspect drug from the EV database. Demographic data (patients aged 18–64 years, female-sex predominant) were consistent with previously reported studies [30]. From 2008 until 2020, there was an increase in ICSR reporting for FIL and PEG-F with the highest reporting taking place in 2013. The increase in reporting is mainly attributed to the increased use of FIL and PEG-F in hospital settings. Indeed, various biosimilars of FIL have gained approval in 2008, becoming increasingly available for clinical use and since then, their demand has constantly increased [24].

Studying ICSR information on seriousness, 89.8% of cases were serious and mainly attributed to PEG-F. As for safety, comparing once-per-cycle PEG-F and daily injections of FIL, a phase III study revealed that the similar ADR profile between FIL and PEG-F [17,31]. Additionally, a meta-analysis study performed by authors identified 13 studies showing a head-to-head comparison of PEG-F and FIL [32]. In nine randomized controlled trials (RCTs), FN-related relative risk was lower for PEG-F and was not statistically significant (RR 0.90; *p* = 0.42). Another meta-analysis also showed statistically insignificant data (0.86; *p* = 0.226) among 12 of 36 RCTs, with lower FN incidence for PEG-F versus FIL. However, 24 of 36 non-RCTs non-randomized controlled trials (NRCTs) showed significantly lower risk with PEG-F than FIL for FN incidence (0.67; *p* = 0.023) [33]. The greater efficacy of PEG-F in NRCTs might be because of the under dosing of FIL (11 out of 12 RCTs provided greater than or equal to 7 doses of FIL versus only 2 of 24 NRCTs). When dosed homogeneously, i.e., a single dose of PEG-F and 10 to 14 days of FIL administration, the ADRs were shown to be similar [34].

Lastly, in line with previously published literature, we observed the frequent reporting of the following ADRs: pyrexia, febrile neutropenia, bone pain and back pain [35,36]. These reactions occur at neutropenia grade 1 to 2 and recovered quickly [37]. The odds ratio with ADRs belonging to the SOC “Investigations” showed no difference between FIL and PEG-F (1.01; 95% CI 0.93–1.01). An increase in the ADRs of the SOC “General disorders and administration site conditions” (1.14; 95% CI 1.06–1.21) and the SOC “Musculoskeletal and connective tissue disorders” (1.21; 95% CI 1.18–1.32) were observed with PEG-F as compared with FIL. The results of a meta-analysis of 5 RCTs (evaluating 617 patients) administered with single dose of PEG-F per cycle compared to daily FIL injections showed that the incidence of bone pain (0.95; 95% CI 0.76–1.19) was similar for FIL and PEG-F and our study confirmed the same. Additionally, in statistical testing, the Q value was not statistically significant indicating that statistical heterogeneity did not occur in the combined studies [38].

As compared with VigiBase^®^, an earlier study performed by the authors, the analysis in the EV database yielded more ICSRs, which may be because different countries adopt different strategies to the execution of these databases, for example, the timelines and typology of reports, definition of ADRs by HCPs and differences in the database management. The analysis on ADRs associated with FIL and PEG-F in the EV database provided an update on the published literature. The overall identified ICSRs were low and are increasing, reflecting increased consciousness among the HCPs.

The present study analyzed the safety profile of FIL and PEG from the spontaneous voluntary data obtained from the EV database. Considering the recent patent expiry of FIL and PEG-F and the approval of their respective biosimilars, our study epitomizes the first complete evaluation of pharmacovigilance data related to FIL and PEG-F. Furthermore, the data from spontaneous reporting, especially in the elderly and patients with co-morbidities, were obtained, as these patients are usually excluded in pre-marketing clinical trials. The European database allowed us to access the data from Europe of FIL and PEG-F.

However, despite its strengths, the study has a few limitations. The ADRs were reported spontaneously by the reporter in specified forms. Thus, the completeness of the reporting forms and the authenticity of data are non-heterogenous and only rely on the accuracy of the reporter. Additionally, only the numerator information on ICSRs was present, but the number of patients exposed to FIL and PEG-F is lacking. Therefore, it is difficult to quantify the overall risk associated with the drugs. The spontaneous reporting system also has the limitation of reporting bias. The authors reported the content mentioned in the spontaneous reports and the evaluation was not modified. This is because the authors did not have any access to the patients’ medical records, but only to the data provided by the source in the reporting form. Thus, spontaneous reporting is mainly affected by the under-reporting and incompleteness of information. An important limitation of the database is the impossibility to compute and provide a distinction between the FIL and PEG-F innovators and the biosimilars. Therefore, the reports of the innovator and biosimilars cannot be separated.

## 5. Conclusions

The descriptive analysis of safety reports related to FIL and PEG-F were carried out using the EV database. Our results demonstrated the similar occurrence of bone pain in patients administered with FIL and PEG-F. For the incidence of pyrexia and back pain, PEG-F was associated with a higher reporting probability as compared to FIL. However, the incidence of neutropenia and febrile neutropenia was higher in FIL compared to PEG-F. Additionally, the majority of the reports were serious and belonged to females. The safety of drugs cannot be concluded from clinical trials because of limited patient exposure and low follow-up with patients. Thus, spontaneous reporting of ADRs, despite of their limitations, is the main source of reporting. The adverse reactions reported with FIL and PEG-F g may affect the financial burden of treatment and may have impact on patients’ quality of life. In this context, a pharmacovigilance system guarantees the continuous monitoring of the safety of drugs and plays an essential role in accessing the benefit risk profile of these drugs.

## Figures and Tables

**Table 1:** Study of common adverse effects between filgrastim and pegfilgrastim.

System Organ Class	PT Term	Filgrastim ICSRs (*n* = 7091)	Pegfilgrastim ICSRs (*n* = 10,312)
General disorders and administration site conditions (*n* = 6084)		[*n* = 2600]	[*n* = 3484]
	Pyrexia	7.8% (559)	6.8% (705)
	Drug Ineffectiveness	5.7% (407)	5.4% (559)
	Death	3.4% (244)	5.3% (548)
Blood and lymphatic system disorders (*n* = 4908)		[*n* = 1766]	[*n* = 3142]
	Neutropenia	8.1% (579)	10.9% (1129)
	Febrile Neutropenia	7.4% (526)	13.4% (1391)
	Thrombocytopenia	3.1% (221)	1.9% (199)
Musculoskeletal and connective tissue disorders (*n* = 2662)		[*n* = 1191]	[*n* = 1471]
	Bone Pain	6.6% (473)	6.9% (719)
	Back Pain	4.2% (303)	2.3% (240)
	Arthralgia	3.0% (214)	2.5% (267)
Investigations (*n* = 2740)		[*n* = 1123]	[*n* = 1617]
	WBC count decreased	3.2% (229)	3.3% (346)
	Platelet count decreased	2.1% (155)	1.3% (135)
	Neutrophil count decreased	1.7% (123)	2.3% (244)

**Table 2:** Reporting odds ratio of ICSRs with ADRs belonging to the SOCs “General disorders and administration site conditions”, “Blood and lymphatic system disorders”, “Musculoskeletal and connective tissue disorders” and “Investigations”, for the comparison of filgrastim versus pegfilgrastim.

ICSRs	ROR (95% CI)	*p* Value
General disorders and administration site conditions	1.14 (1.06–1.21)	<0.0001
Blood and lymphatic system disorders	0.75 (0.70–0.80)	<0.0001
Musculoskeletal and connective tissue disorders	1.21 (1.18–1.32)	<0.0001
Investigations	1.01 (0.93–1.10)	0.78

Abbreviations: ICSRs = Individual Case Safety Reports; ADRs = adverse drug reactions; SOCs = System Organ Classes; ROR = reporting odds ratio; CI = confidence interval.

**Table 3:** Reporting odds ratio of ICSRs with ADRs belonging to the top reported ADRs for the comparison of filgrastim versus pegfilgrastim.

ADRs	ROR (95% CI)	*p* Value
Pyrexia	1.16 (1.04–1.30)	<0.05
Bone Pain	0.95 (0.84–1.07)	0.456
Back Pain	1.87 (1.57–2.22)	<0.0001
Neutropenia	0.72 (0.65–0.80)	<0.0001
Febrile Neutropenia	0.51 (0.46–0.57)	<0.0001

Abbreviations: ADRs = adverse drug reactions; ROR = reporting odds ratio; CI = confidence interval.

## Data Availability

The data generated from the study are clearly presented and discussed in the manuscript.
